# Ethyl Acetate Fraction of *Lannea microcarpa* Engl. and K. Krause (Anacardiaceae) Trunk Barks Corrects Angiotensin II-Induced Hypertension and Endothelial Dysfunction in Mice

**DOI:** 10.1155/2019/9464608

**Published:** 2019-04-28

**Authors:** Mathieu Nitiéma, Raffaella Soleti, Camille Koffi, Lazare Belemnaba, Patricia Mallegol, Noufou Ouédraogo, Félix Bondo Kini, Sylvin Ouédraogo, Innocent Pierre Guissou, Ramaroson Andriantsitohaina

**Affiliations:** ^1^Institut de Recherche en Sciences de la Santé, Centre National de la Recherche Scientifique et Technologique (IRSS/CNRST), 03 BP 7192 Ouagadougou 03, Burkina Faso; ^2^Laboratoire du Développement du Médicament, Université Ouaga I Pr Joseph KI-ZERBO, 03 BP 7021 Ouagadougou 03, Burkina Faso; ^3^SOPAM, U1063, INSERM, Univ Angers, SFR ICAT, Bat IRIS-IBS, Rue des Capucins, 49100 Angers, France

## Abstract

Traditional remedies prepared from *Lannea microcarpa* leaves, barks, roots, and fruits are used to treat many diseases including hypertension. This study investigated whether oral administration of the ethyl acetate fraction of *Lannea microcarpa* trunk barks (LMAE) corrects angiotensin (Ang) II-induced hypertension in mice. Its effects on vascular function were specifically investigated. Experiments explored hemodynamic and echocardiographic parameters *in vivo* and vascular reactivity to acetylcholine (ACh) and CaCl_2_ *ex vivo* on isolated aortas. Mice received LMAE for 3 weeks (50 mg/kg/day) by oral gavage. In the last two weeks of treatment, mice were implanted with osmotic minipumps delivering NaCl (0.9%) or Ang II (0.5 mg/kg/day). LMAE completely prevented the increase in systolic and diastolic blood pressure induced by Ang II. Echocardiographic and kidney parameters were not affected by the different conditions. LMAE abrogated Ang II-induced impairment of ACh-induced relaxation without affecting that of sodium nitroprusside. LMAE also completely prevented CaCl_2_-induced contraction in KCl-exposed aorta *ex vivo*. The extract alone did not modify superoxide (O_2_^−^) and nitric oxide (NO^·^) production in femoral arteries from control mice but significantly limited Ang II-induced O_2_^−^ production. These effects were associated with reduced expression of inducible isoform of cyclooxygenase- (COX-) 2 and nicotinamide adenine dinucleotide phosphate (NADPH) oxidase isoform NOX-2 in aortas. Finally, phytochemical analysis showed that LMAE contains sterols, triterpenes, coumarins, and anthraquinone. These results showed that LMAE prevents Ang II-induced hypertension and vascular dysfunction through a reduction of oxidative stress linked to COX-2 and NOX-2 pathway and inhibition of calcium entry. This study provides pharmacological basis of the empirical use of *Lannea microcarpa* trunk bark extract against hypertension.

## 1. Introduction

Hypertension is one of the most widespread and modifiable risk factors for cardiovascular disease worldwide [1, 2]. Despite improvements of antihypertensive treatments, 20-30% of patients with hypertension are resistant to at least three blood pressure-lowering drugs [3, 4]. The use of nonpharmacological treatments to lower blood pressure has been growing in the recent years and especially the administration of nutraceutical supplements based on botanicals and traditional medicine and pharmacopoeia [5].

*Lannea microcarpa*, commonly known as African grape, is widely distributed in the sub-Saharan region and is known for its medicinal properties. Leaf, bark, root, and fruit preparations from *Lannea microcarpa* are traditional remedies used to treat many conditions including mouth blisters, rheumatism, sore throats, dysentery, conjunctivitis, stomatitis, skin eruptions, ulcers, and high blood pressure [6, 7].

Pharmacologically, trunk bark extract of *Lannea microcarpa* has antioxidant activity [8–10] and can mitigate the damaging effects of oxidative stress on cells [11]. *Lannea microcarpa* extracts have also anti-inflammatory properties [12] and induced vascular relaxation *via* the inhibition of phosphodiesterases [13].

Physiologically, the renin-angiotensin system (RAS) is a master regulator of blood pressure and cardiovascular control of neural and endocrine functions. Moreover, the RAS participates in the onset and the evolution of cardiovascular-related diseases including hypertension, heart failure, chronic kidney disease, coronary artery disease, and stroke [14, 15]. The main mediator peptide of RAS is angiotensin II (Ang II) [16, 17] which activates AT1 receptors (AT1R) directly in the vascular smooth muscle leading to strong vasoconstriction. Sustained activation of ATIR leads to anion superoxide (O_2_^−^) overproduction from NADPH oxidase which in turn is responsible for the reduction of bioavailability of the endothelium-derived nitric oxide (NO^·^) and then for the endothelial dysfunction [18, 19]. Also, Ang II stimulates the synthesis and the secretion of aldosterone and consequently alters the absorption of renal sodium and water [20, 21]. One of the best experimental models of human hypertension is the chronic infusion of Ang II. This model displays an increased blood pressure, an impaired endothelium-dependent relaxation, and vascular inflammation [22]. The present study was designed to investigate whether oral administration of the ethyl acetate fraction of *Lannea microcarpa* trunk barks (LMAE) corrects Ang II-induced hypertension in mice, with a specific interest on its effects on vascular functions.

## 2. Materials and Methods

### 2.1. Plant Material

The *Lannea microcarpa* Engl. and K. Krause (Anacardiaceae) trunk barks were collected in January 2012 in the area of Loumbila (zone of savannah), located at 20 km in the northeast of Ouagadougou (Burkina Faso). The plant samples were authenticated at “Herbier National du Burkina (HNBU)” located at the “Département Environnement et Forêt/Centre National de la Recherche Scientifique et Technologique” (DEF-CNRST), Ouagadougou (Burkina Faso), where the voucher specimen had been deposited under number HNBU 361.

The collected sample was air-dried, deprived of solar light and dust, and was powdered using a mechanical grinder. The resulting powder was used for preparation of extracts for antioxidant and biological investigations.

### 2.2. Preparation of the Lyophilized Aqueous Decoction and Fractions with Dichloromethane and Ethyl Acetate

One hundred grams of powder of stem barks of *Lannea microcarpa* was extracted by decoction using 500 mL water distilled during 30 min. The aqueous solution was filtered and then centrifuged at 650×*g* for 5 min. The supernatant was lyophilized (14.78 g) for the various tests. Another preparation of the same concentration was used for fractionation.

The sequential extraction method with the aqueous decoction was performed with two organic solvents based on their polarity. A fractionation from the aqueous decoction (100 mL) was carried out starting with dichloromethane and then ethyl acetate. Exhaustion by the dichloromethane (3 × 50 mL) and ethyl acetate (3 × 50 mL) followed by dry evaporation (35°C) led to the fractions with dichloromethane (LMDCM, 25.2 mg) and with ethyl acetate (LMAE, 457.5 mg), respectively, used for pharmacological, antioxidant, and phytochemical investigations. LMAE was used in the present study because it represented the most effective extract in inducing vascular relaxation, in terms of pD_2_ in the mice aorta and pig coronary artery compared to the aqueous decoction extracts (data not shown).

The dose of LMAE used in the present report was based on our preliminary studies (unpublished data). Invasive blood pressure measurements in rats showed that the 80% of effective dose of LMAE able to reduce hypertension was 0.5 mg/kg. Because it is assumed that only 1% of total compounds could be absorbed in the digestive tract, the dose used was then 50 mg/kg/day.

### 2.3. Ethics Statement

The procedure followed in the present study was approved by the local ethics committee (“Comité d'éthique en expérimentation animale Pays de la Loire”; CEEA.2011.40) in agreement with the guidelines and authorization with the French Ministry of Agriculture regulations based on the European Community.

### 2.4. Animals

Four groups of 8 male Swiss mice (6 to 8 weeks old and weighing 32 to 36 g) were used: (i) group receiving infusion of saline by an osmotic pump for 2 weeks (NaCl), (ii) group receiving Ang II (0.5 mg/kg/day, Sigma-Aldrich; St. Quentin Fallavier, France) by an osmotic pump for the 2 weeks (Ang II) [23], (iii) group receiving LMAE (50 mg/kg body weight/day, oral gavage, suspended in 2% of Tween 80 solution) during 3 weeks and infusion by an osmotic pump for the 2 last weeks (NaCl+LMAE), and (iv) group receiving LMAE (50 mg/kg body weight/day, oral gavage, suspended in 2% of Tween 80 solution) during 3 weeks and Ang II by an osmotic pump for the last 2 weeks (Ang II+LMAE).

Ang II and NaCl were delivered *via* unprimed osmotic minipumps (Model 1002, Alzet Osmotic Pumps, Cupertino, CA, USA) that were subcutaneously implanted into the back of mice (details of the surgical procedure were given in Supplemental Materials). All experiments were conducted in mice housed in a temperature-controlled animal facility with a 12-hour light/dark cycle and free access to rodent chow and tap water. At the end of the protocol, mice were euthanized. The blood, heart, kidneys, aorta, and femoral artery were collected for further examinations.

### 2.5. Blood Pressure and Heart Rate Measurements

Noninvasive blood pressure (systolic and diastolic) and heart rate were measured in conscious mice using the tail-cuff plethysmography system (Letica, Barcelona, Spain). All mice were trained with the device to accustom them to the procedure for 1 week prior to the start of the protocol. For blood pressure and heart rate determination, 6 consecutive measurements were obtained daily and averaged.

### 2.6. Echocardiography Examination

Cardiac function was measured as previously described [24]. Briefly, transthoracic echocardiography was performed on anesthetized (1.5% isoflurane) mice using the Vevo 770 ultrasound echograph from FUJIFILM VisualSonics (Toronto, ON, Canada) with a 30 MHz imaging transducer. Parasternal short-axis images were obtained in M-mode. Systolic and diastolic diameters, stroke volume, cardiac output, ejection and fractions, and left ventricle posterior and anterior wall (LVPW, LVAW) thickness in the systolic and diastolic phase were evaluated.

### 2.7. Biochemical Parameters

At the end of the protocol, blood was collected and centrifuged at 4°C for 10 min at 900×*g*. Plasma samples were frozen in liquid nitrogen and stored at -80°C until assayed. Biochemical analyses were performed with plasma using a Konelab™ 20 Clinical Chemistry Analyzer (Thermo Scientific™, Waltham, MA, USA) by assaying sodium (Na^+^), chloride (Cl^−^), urea and creatinine.

### 2.8. Myography

*Ex vivo* vasorelaxation experiments were conducted on thoracic aorta according to the method previously described [25]. Upon mouse euthanasia, the thoracic aorta was removed and pinned in a dissecting dish and cleaned of fat and connective tissue. Segments of the aorta (2 mm in length) were mounted on myographs (Danish Myo Technology, Aarhus, Denmark) filled with physiological salt solution (PSS). The composition of PSS (in mM) was 130 NaCl, 14.9 NaHCO_3_, 3.7 KCl, 1.2 MgSO_4_·7H_2_O, 1.6 CaCl_2_·H_2_O, 1.2 KH_2_PO_4_, and 11 glucose. The PSS was continuously kept at 37°C and aerated with a gas mixture of 95% O_2_ and 5% CO_2_ at pH 7.4. Endothelium-dependent vasodilatation was evaluated by cumulative addition of Ach (1 nM-10 *μ*M, Sigma-Aldrich) in order to construct a concentration-response curve on aortic rings precontracted with the thromboxane analogue A2 agonist, 9,11-dideoxy-9*α*,11*α*-methanoepoxy PGF2*α* (U46619, Merck Chemicals Ltd., Nottingham, UK) (80% of the maximal contractile response), as previously described [23].

Concentration-response relaxation to sodium nitroprusside (SNP, 1 nM-10 *μ*M, Sigma-Aldrich) was also studied after precontraction of the aortas with U46619 (80% of the maximal contractile response).

In another set of experiment, the effects of LMAE on calcium-induced contractile responses of CaCl_2_-exposed mice vessels were conducted on Swiss mice (healthy, untreated mice) thoracic aorta.

The presence of functional endothelium was assessed by the ability of ACh (10 *μ*M, Sigma-Aldrich) to induce more than 80% relaxation of vessels precontracted with U46619. In some rings, the endothelium was denuded by gently rubbing the intimal space with forceps. Endothelium-denuded aorta ring was considered effectively removed when 10 *μ*M of ACh caused less than 10% relaxation. After testing the response of the vessels to PSS containing KCl 134 mM, CaCl_2_, H_2_O 1.6 mM, and without NaCl (indicated concentrations of KCl substituted for equimolar amounts of NaCl, 130 mM). The bathing solution was replaced by a “calcium-free” depolarizing medium (Ca_0_-KCl PSS; this PSS contains 80 mM KCl). Each preparation was exposed to Ca_0_-KCl PSS concentration. After a 45 min washout period, cumulative additions of CaCl_2_ (10^−5^ to 10^−2^ M) were repeated two times, separated by 60 min washout periods, and consecutive concentration-response curves constructed. When LMAE (500 *μ*g/mL) was used, it was added 5 min before the cumulative addition of CaCl_2_ on vessels exposed to Ca_0_-KCl PSS [26]. LMAE was prepared freshly in distilled water and DMSO (Sigma-Aldrich) with a final concentration of 0.02% of DMSO. After each experiment, the ring length was measured using a micrograduated magnification eyepiece.

### 2.9. Superoxide (O_2_^−^) and NO^·^ Spin Trapping by Electron Paramagnetic Resonance (EPR) Studies

The method for O_2_^–^ detection was previously described [24]. The femoral arteries isolated from all mice were dissected and allowed to equilibrate in deferoxamine-chelated Krebs-Hepes solution containing 1-hydroxy-3-methoxycarbonyl-2,2,5,5-tetramethylpyrrolidin (CMH; 500 mM, Noxygen, Mainz, Germany), deferoxamine (25 mM, Sigma-Aldrich), and diethyldithiocarbamate (DETC; 5 mM, Sigma-Aldrich) at 37°C) for 45 min. The arteries were then frozen using liquid nitrogen.

NO^·^ detection was performed using DETC as a spin trap as previously described [24]. The isolated femoral arteries were incubated for 45 min in a solution containing Krebs-Hepes buffer (BSA, 20.5 g/L, Sigma Aldrich), CaCl_2_ (3 mM), and L-arginine (0.8 mM, Sigma-Aldrich). Diethyldithiocarbamate-iron(II) complex (Fe[DETC]_2_) solution was added to the vessel and incubated for 45 min at 37°C. The arteries were then immediately frozen in plastic tubes using liquid nitrogen.

Both O_2_^−^ and NO^·^ measurements were performed on a table-top x-band spectrometer miniscope (MS200; Magnettech, Berlin, Germany). Recordings were made at 77°K, using a Dewar flask. Instrument settings were 10 mW of microwave power, 1 mT of amplitude modulation, 100 kHz of modulation frequency, 180 s of sweep time, and 4 scans. Values are expressed as the amplitude of signal per mg weight of dried femoral artery.

### 2.10. Western Blotting

Aorta samples were frozen in liquid nitrogen and homogenized in buffer containing 500 *μ*L sodium dodecyl sulfate (SDS, 20%), 100 *μ*L sodium orthovanadate, 50 *μ*L Na-pyrophosphate, 200 *μ*L Tris-HCl 500 mM (pH 7.4), and 400 *μ*L antiprotease. The suspensions were centrifuged at 15,000×*g* for 15 min at 4°C. Supernatants containing the proteins were collected and stored at -80°C until use. Proteins (40 *μ*g) were separated using 4-12% sodium dodecyl sulfate-polyacrylamide gel electrophoresis (SDS-PAGE, Invitrogen, Carlsbad, CA). After electrophoresis, proteins were transferred to nitrocellulose membranes and the membrane were then saturated at room temperature for 1 h in TBS-T (20 mM Tris base, 61.5 mM NaCl pH 7.8, and 0.1% Tween 20) buffer containing 5% BSA. The membrane was incubated with primary antibody for 2 h at room temperature. The murine polyclonal antibody for cyclooxygenase 1 (COX-1, 1 : 1000, Santa Cruz Biotechnology, Dallas, TX), mouse monoclonal antibody for cyclooxygenase 2 (COX-2, 1 : 500, BD Pharmingen, San Jose, CA), mouse monoclonal antibody for NADPH oxidase 2 (NOX-2: gp91-phox, 1 : 1000, Santa Cruz Biotechnology), and goat polyclonal antibody for NADPH oxidase 4 (NOX-4, 1 : 1000, Santa Cruz Biotechnology) were used. The same membrane was used to determine *β*-actin expression (loading) control using a polyclonal anti-mouse *β*-actin antibody (1 : 5000, Sigma Aldrich). The membrane was then incubated for 90 min at room temperature with the horseradish peroxidase- (HRP-) conjugated secondary antibody. Membranes were washed at least three times in Tris buffer solution containing 0.05%. The bands were visualized using the enhanced chemiluminescence system and quantified by densitometry. Images analysis were performed using ImageJ software (National Institute of Mental Health, Bethesda, Maryland, USA).

### 2.11. DPPH^·^ Assay

The radical scavenging activity was performed using 2,2-diphényl-1-picrylhydrazyl (DPPH, Sigma-Aldrich) as previously described [27, 28]. Briefly, the absorbance of 10 *μ*L of samples, standard, and Trolox (Sigma-Aldrich) added to 200 *μ*L of DPPH was measured at 490 nm after 30 min incubation in the dark at room temperature using a Bio-Rad spectrophotometer (Model 680, Japan). The result was expressed as Trolox (Sigma-Aldrich) equivalent antioxidant capacity according to the following equation: TEAC = extract antiradical power (ARP)/Trolox ARP, where ARP was the amount of antioxidant necessary to decrease the initial DPPH^·^ concentration by 50% (ARP = 1/IC50).

### 2.12. Ferric Reducing Antioxidant Power (FRAP) Assay

FRAP assay was performed in extract, fractions, and Trolox as previously described [29]. The mixture of 0.5 mL of samples with 1.25 mL of phosphate buffer and 1.25 mL of aqueous solution of potassium hexacyanoferrate (1%, Prolabo, Paris, France) was incubated for 30 min at 50°C. Then, 1.25 mL of trichloroacetic acid (10%, Sigma-Aldrich) was added and centrifugated at 3000×*g* for10 min. Distilled water (0.625 mL) and FeCl_3_ solution (0.125 mL, 0.1%) were added to the upper layer solution (0.625 mL), and the absorbance was measured at 700 nm using a spectrophotometer (Agilent, Santa Clara, CA) equipped with UV-visible ChemStation software. Trolox was used to the plot calibration curve. FRAP activity of samples was expressed in mmol Trolox equivalent/gram of dry extract.

### 2.13. Phytochemical Screening by Liquid Medium

Phytochemical screening of the decoction and fractions of stem barks of *Lannea microcarpa* was conducted following the Ciulei method [30]. Phytochemical groups were determined in the different samples using the following characterization tests: iron chloride test, Shibata test, Liebermann-Büchard test, foam index, and fluorescence to the UV lamp 365 nm for identification of tannins, flavonoids, sterols and triterpenes, saponins, and coumarins, respectively. Borntranger's test was used for the detection of anthraquinones and emodols. Alkaloids were characterized by the reactions of Dragendorff and Mayer. The reducing compounds were characterized by the reaction of the Fehling reagent. For the anthocianosides tests, 1-2 sodium hydroxide pellets were added to 1 mL of extract with the appearance of a blue color the presence of anthocyanins in the extract.

### 2.14. Phytochemical Analysis of Extracts by Thin-Layer Chromatography (TLC)

The LMAE fraction was loaded on Silica gel 60 F_254_ plates (Merck). The elution was carried out using two solvent systems: (i) n-hexane/ethyl acetate/toluene (3/1/1 *v*/*v*/*v*) was used to migrate anthocianosides, coumarins, sterols and triterpenes, and tannin compounds and (ii) ethyl acetate/formic acid/distilled water (6/1/1 *v*/*v*/*v*) was used to migrate saponin compounds.

Samples (10 mg/mL, 10 *μ*L) were directly loaded as spot into the TLC plates. The coumarin compounds were observed under a UV lamp (254/366 nm) after spraying with a specific developer. The developer 5% KOH in 95% methanol was used to show the presence of anthracenosids and coumarins. Anisaldehyde-sulfuric acid was used to detect saponins, and 2% FeCl_3_ in 95% methanol was used to detect sterols, triterpenes, and tannins.

### 2.15. Statistical Analysis

Data were analyzed using GrapPad Prism 5.02 software (GrapPad Software, San Diego, CA, USA). The results are expressed as mean ± SEM. For animal experiments, *n* represented the number of mice. Blood pressure, heart rate, and myography experiment values were compared using a two-way ANOVA with Bonferroni post hoc test. Data of western blot and electron paramagnetic resonance experiments as well as DPPH^·^ and FRAP assays were compared using one-way ANOVA followed by a Bonferroni post hoc test. ^∗^ *p* < 0.05 was considered to be significant.

## 3. Results

### 3.1. Blood Pressure and Heart Rate

**Figure 1:**
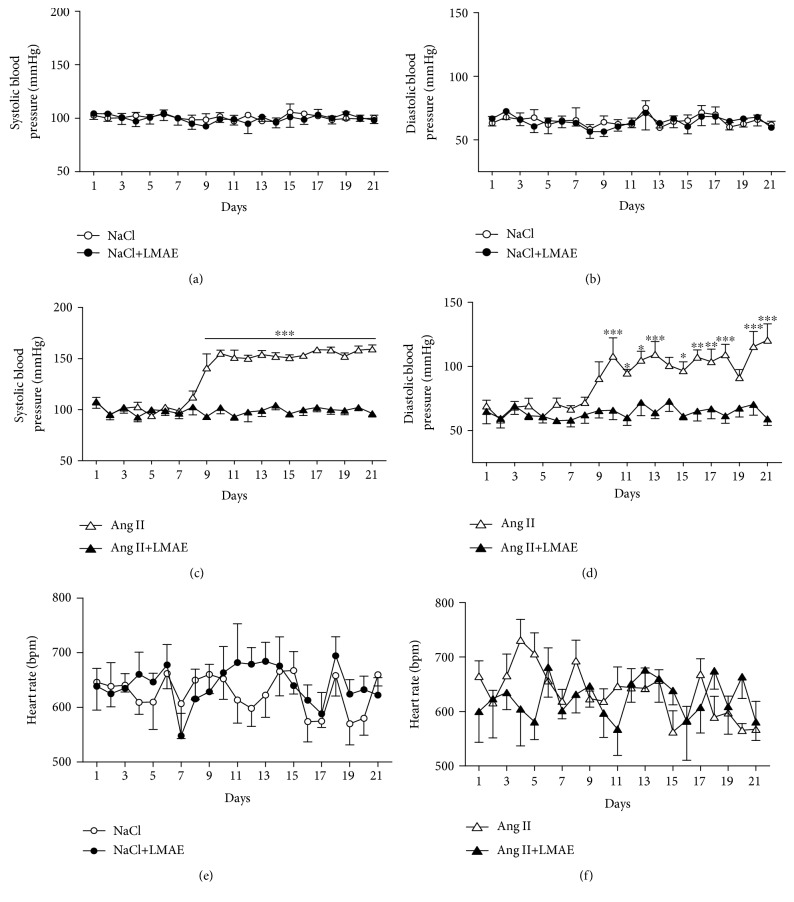
Effects of LMAE on systolic and diastolic blood pressure and heart rate in an Ang II-induced hypertension model. LMAE (50 mg/kg) was administered daily by oral gavage for 3 weeks, and osmotic minipumps delivering NaCl (0.9%) or angiotensin II (0.5 mg/kg/day) were implanted 1 week after the start of the LMAE pretreatment. The effect of LMAE was studied in control mice (a, b, and e) and Ang II-treated mice (c, d, and f). Systolic blood pressure (a, c) and diastolic blood pressure (b, d) were measured daily as well as the heart rate (e, f). Angiotensin II (Ang II) treatment induced hypertension that was prevented by LMAE. NaCl: mice treated with NaCl, control; NaCl+LMAE: mice treated with LMAE and NaCl; Ang II: mice treated with Ang II; Ang II+LMAE: mice treated with LMAE and Ang II. NaCl and Ang II groups were treated with an equal volume of vehicle (2% of Tween 80). Results are given as means ± SEM. *n* = 7-8/group; ^∗^ *p* < 0.05; ^∗∗^ *p* < 0.01; ^∗∗∗^ *p* < 0.001.

In mice receiving saline alone and saline (NaCl 0.9%) plus LMAE (Figures 1(a) and 1(b)), systolic blood pressure and diastolic blood pressure were stable throughout the experiment. As expected, Ang II increased the systolic and diastolic blood pressure, which became significant at day 8. In the other group of mice, LMAE completely prevented Ang II-induced hypertension (Figures 1(c) and 1(d)). Also, independently from treatment, heart rate values were not modified in any group of mice throughout the experiments (Figures 1(e) and 1(f)).

### 3.2. Heart and Kidney Function

Cardiac parameters measured by echocardiography (Table 1) were not affected by the different treatments throughout the study.

No significant changes in heart and kidney weights were observed in all groups of mice (Supplemental Figures 1A and 1B). Also, neither LMAE nor Ang II affected kidney markers (creatinine, urea) and electrolytes (Na^+^ and Cl^−^) in all groups of mice (Supplemental Figure 1C–1F).

### 3.3. Vascular Function

**Figure 2:**
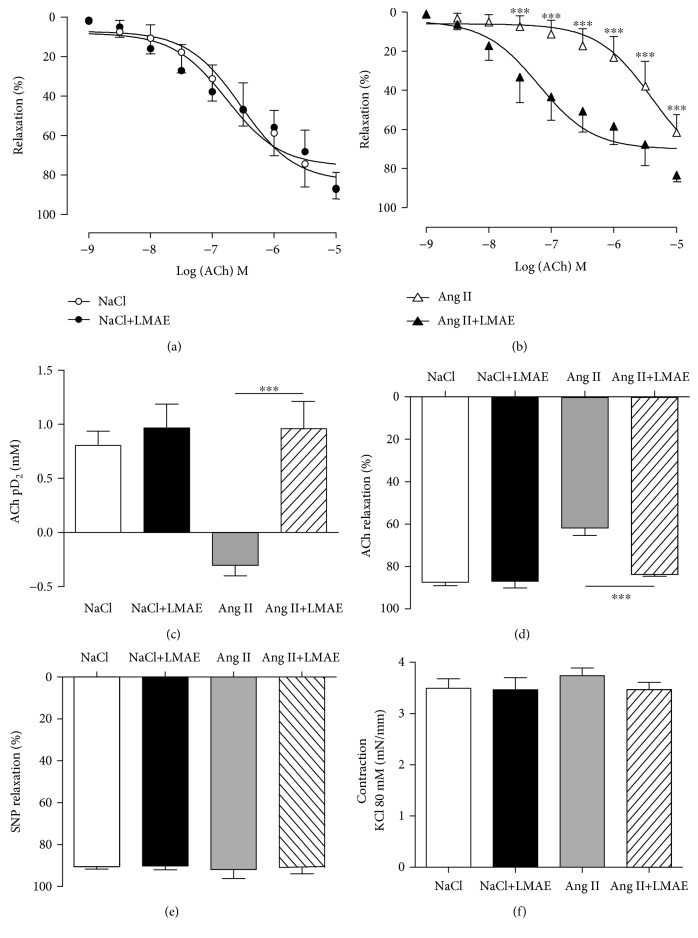
Effect of LMAE on vascular function *ex vivo*. LMAE (50 mg/kg) was administered daily by oral gavage for 3 weeks, and osmotic minipumps delivering NaCl (0.9%) or angiotensin II (0.5 mg/kg/day) were implanted 1 week after the start of the LMAE pretreatment. The effect of LMAE on Ach-induced vasorelaxation was studied in aortic rings precontracted with U46619 isolated from control mice (a) and Ang II-treated mice (b). Sensitivity to acetylcholine represented by pD2 (c) and maximal effect (Emax) (d) were measured. The same aortic rings were relaxed with SNP, Emax (e); after precontraction with KCl (80 mM), Emax (f). NaCl: mice treated with NaCl, control; NaCl+LMAE: mice treated with LMAE and NaCl; Ang II: mice treated with Ang II; Ang II+LMAE: mice treated with LMAE and Ang II. NaCl and Ang II groups were treated with an equal volume of vehicle (2% of Tween 80). The results are expressed as mean ± SEM. *n* = 7-8/group; ^∗∗∗^ *p* < 0.001.

ACh-induced endothelium-dependent relaxation was significantly reduced in the aortas of Ang II-treated mice compared to other groups of mice. LMAE extract prevented Ang II-induced endothelial dysfunction (Figures 2(a) and 2(b)). LMAE prevented both the decreased sensitivity (pD_2_) and reduced maximal relaxation (Emax) to ACh induced by Ang II (Figures 2(c) and 2(d)) with a relaxation profile similar to the control groups (NaCl), LMAE (NaCl+LMAE).

Relaxation to SNP and vasoconstriction induced by 80 mM KCl were similar in the four groups studied (Figures 2(e) and 2(f)).

### 3.4. O_2_^−^ and NO^·^ Production in the Femoral Artery

**Figure 3:**
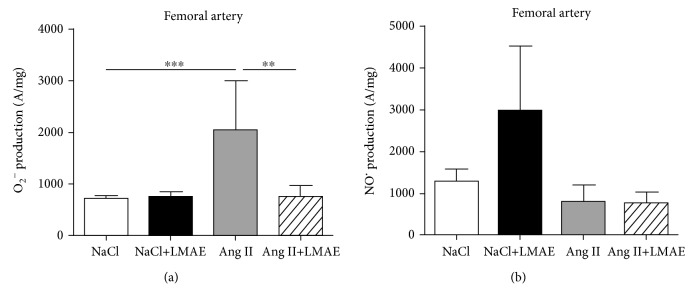
Effects of LMAE on superoxide and nitric oxide production *ex vivo*. LMAE (50 mg/kg) was administered daily by oral gavage for 3 weeks, and osmotic minipumps delivering NaCl (0.9%) or angiotensin II (0.5 mg/kg/day) were implanted 1 week after the start of the LMAE pretreatment. Nitric oxide (NO^·^) (a) and superoxide (O_2_^−^) (b) production was measured in femoral arteries. NaCl: mice treated with NaCl, control; NaCl+LMAE: mice treated with LMAE and NaCl; Ang II: mice treated with Ang II; Ang II+LMAE: mice treated with LMAE and Ang II. NaCl and Ang II groups were treated with an equal volume of vehicle (2% of Tween 80). The results are expressed as mean ± SEM. *n* = 7-8/group; ^∗^ *p* < 0.05.

LMAE did not affect the O_2_^−^ level in the femoral artery of control animals. Ang II treatment significantly increased the O_2_^−^ level. This was completely prevented by LMAE treatment (Figure 3(a)).

NO^·^ production in the femoral artery was not statistically different in the four groups studied, although LMAE seemed to slightly increase its production in control vessels (Figure 3(b)).

### 3.5. NADPH and COX Pathway Evaluation

**Figure 4:**
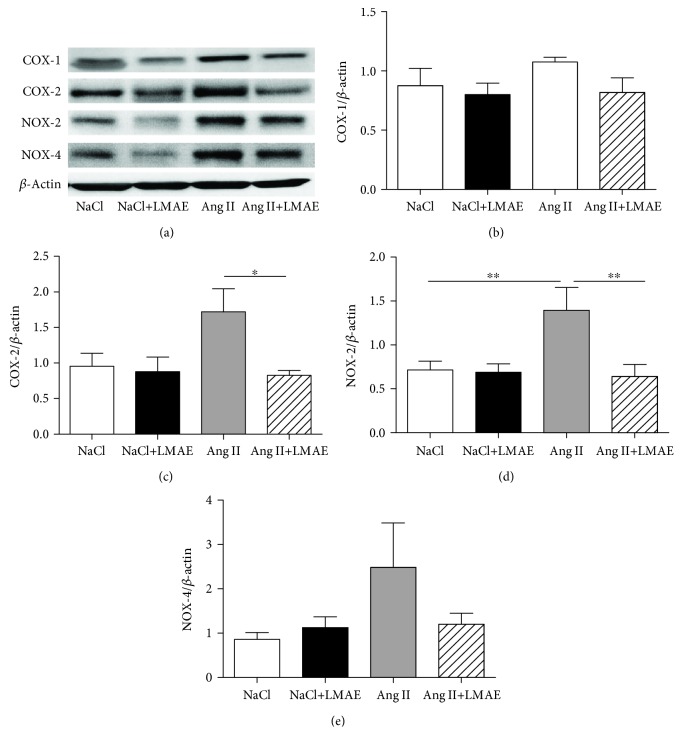
Effects of LMAE on the protein expression of prooxidant enzymes *ex vivo*. LMAE (50 mg/kg) was administered daily by oral gavage for 3 weeks, and osmotic minipumps delivering NaCl (0.9%) or angiotensin II (0.5 mg/kg/day) were implanted 1 week after the start of the LMAE pretreatment. The protein expression of prooxidant enzymes was measured in isolated aortas. A representative image of the blots is shown in (a). Quantitative evaluation of COX-1 (b), COX-2 (c), NOX-2 (d), Ang II under grey histogram and NOX-4 (e) protein expressions was performed. NaCl: mice treated with NaCl, control; NaCl+LMAE: mice treated with LMAE and NaCl; Ang II: mice treated with Ang II; Ang II+LMAE: mice treated with LMAE and Ang II. NaCl and Ang II groups were treated with an equal volume of vehicle (2% of Tween 80). The results are expressed as mean ± SEM. *n* = 5-6/group; ^∗^ *p* < 0.05.

Protein expression of enzymes involved in the modulation of reactive oxygen species production, including COX-1, COX-2, NOX-2, and NOX-4, was analyzed (Figure 4(a)). The aortas from Ang II-treated mice displayed a nonsignificant increased expressions of COX-1 and COX-2 compared to those isolated from either NaCl or NaCl+LMAE mice. Interestingly, LMAE treatment significantly decreased COX-2 but not COX-1 expressions in the aortas isolated from Ang II-treated mice compared to those from Ang II-treated mice alone (Figures 4(b) and 4(c)).

Ang II treatment significantly increased aortic expressions of NOX-2 compared to nonhypertensive groups, without affecting NOX-4 (Figures 4(a), 4(d), 4(e)). LMAE treatment prevented Ang II-induced increase of NOX-2 expression in the aorta (Figure 4(d)).

### 3.6. *In Vitro* Characterization of LMAE Antioxidant Activity

**Figure 5:**
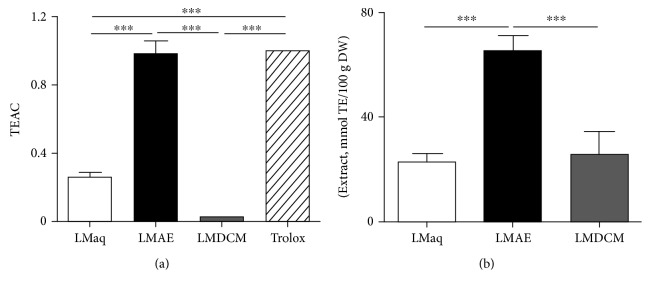
*In vitro* antioxidant activity of *Lannea microcarpa* trunk bark extracts. Antioxidant activity of aqueous decoction extract (LMaq) and its fractions dichloromethane (LMDCM) and ethyl acetate (LMAE) of *Lannea microcarpa* was investigated *in vitro* using DPPH^·^ (a) assay and FRAP assay (b). TEAC: Trolox equivalent antioxidant capacity; TE: Trolox equivalent; DW: dry weight. The results are expressed as mean ± SEM of triplicate; ^∗∗∗^ *p* < 0.001.

Antioxidant activity of aqueous decoction extract (LMaq) and its fractions dichloromethane (LMDCM) and ethyl acetate (LMAE) of *Lannea microcarpa* was investigated *in vitro* using Trolox equivalent antioxidant capacity (TEAC) assay and FRAP assay. LMAE was the most potent antioxidant extract with an antioxidant capacity similar to the standard, Trolox. LMaq had a lower antioxidant capacity compared to LMAE, and LMDCM antioxidant capacity was almost negligible (Figure 5(a)).

The FRAP assay measured the reductive activity of Fe^3+^. LMAE displayed a higher reductive activity compared to LMaq and LMDCM that showed similar effects (Figure 5(b)).

### 3.7. *Ex Vivo* Characterization of LMAE on Vasoconstriction

**Figure 6:**
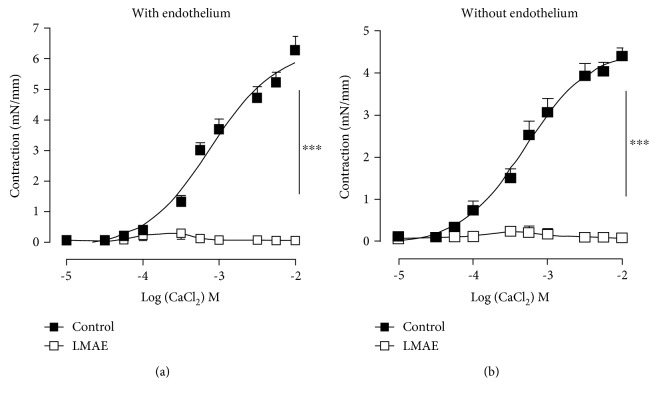
Effect of LMAE on CaCl2-induced contraction *ex vivo*. Cumulative concentrations of CaCl2 (10^−5^-10^−2^ M) were added to aortic rings with intact (a) or denuded (b) endothelium in Ca^2+^-free Krebs solution, containing 80 mM KCl, to activate voltage-dependent calcium channels (VDCCs). Aortic rings were either preincubated without vehicle (0.02% of DMSO) or LMAE (500 *μ*g/mL). The results are expressed as mean ± SEM. (*n* = 5). ^∗∗∗^ *p* < 0.001.

The effect of LMAE was also investigated on calcium-induced contraction ex vivo. Cumulative concentrations of CaCl_2_ (10^−5^-10^−2^ M) were added to aortic rings with intact- or denuded endothelium in Ca^2+^-free Krebs solution, containing 80 mM KCl to activate voltage-dependent calcium channels (VDCCs) (Figure 6). Compared to vehicle (0.02% of DMSO), preincubation with LMAE (500 *μ*g/mL) dramatically inhibited CaCl_2_-induced contraction of the aortas with (Figure 6(a)) and without functional endothelium (Figure 6(b)).

### 3.8. Phytochemical Characterization of *Lannea microcarpa* Trunk Bark Extracts

The phytochemical study of the powder of *Lannea microcarpa* trunk barks showed the presence of steroids and triterpenoids in the dichloromethane fraction. The ethyl acetate fraction showed the presence of anthraquinone, steroids, triterpenoids, and coumarins. The residual aqueous fraction showed the presence of tannins and saponins. The qualitative phytochemical analysis in liquid medium of *Lannea microcarpa* trunk bark extracts is summarized in Table 2. The representation of revealed phytochemical groups by the TLC was illustrated in Supplemental Figure 2.

## 4. Discussion

We report that LMAE completely prevented Ang II-induced hypertension without modification of cardiac nor kidney functions. The beneficial effects of LMAE treatment was associated with improvement of endothelial dysfunction and decrease of arterial O_2_^−^ production. This effect was probably due to the capacity of LMAE to reduce expression of prooxidant enzymes such as COX-2 and NOX-2 in the aorta. It can also be linked to the strong antioxidant properties of LMAE *in vitro*. Finally, LMAE prevented CaCl_2_-induced contraction in the KCl-exposed aorta ex vivo. Altogether, this study established for the first time the mechanism underlying the antihypertensive action of LMAE which seemed to target mainly blood vessels.

Our aim was to provide pharmacological basis for the use of traditional remedies such as the extract of *Lannea microcarpa* trunk barks to treat hypertension in a well-established animal model. This was based on preliminary studies reporting potent antioxidant properties of *Lannea microcarpa* extracts and fractions obtained from the leaves, seed, and fruit. Moreover, LMAE had been shown to induce vasorelaxation of the rat thoracic aorta *via* inhibition of phosphodiesterases [13]. All of these effects may concur to the potential protective effect of LMAE. However, these studies have been performed in normotensive animals and the antihypertensive effect of LMAE was not characterized and the mechanism underlying its actions not completely understood.

In the present report, we demonstrate that LMAE is efficient against Ang II-induced hypertension in mice. Of particular interest was that LMAE did not possess hypotensive property in normotensive mice. LMAE did not affect the cardiac or kidney structure and function in an Ang II-induced hypertension model. These results indicate that the antihypertensive effect of LMAE is mainly due to its vascular action, leading to the reduction of vascular resistance. Correction of endothelial dysfunction has been reported previously in the same experimental model of hypertension using red wine polyphenols [31] or microparticles bearing sonic hedgehog [23]. With regard to red wine polyphenols, prevention of vascular NADPH oxidase induction and preservation of arterial NO^·^ availability during Ang II administration likely contributed to this effect. For microparticles bearing sonic hedgehog, increased NO^·^ production and reduction of oxidative stress concurred to endothelial protection.

We showed that Ang II treatment impaired endothelium-dependent relaxation to Ach and LMAE completely prevented endothelial dysfunction. It is well established that vascular oxidative stress induced endothelial dysfunction most likely by inactivating NO^·^. In the present work, LMAE alone did not modify O_2_^−^ and NO^·^ productions in the femoral arteries but significantly reduced O_2_^−^ in the vessels from angiotensin II-treated mice. Thus, LMAE might exert its protective effect by decreasing oxidative stress. Although we did not observe an increase in NO^·^ production in the vessels of Ang II-treated mice, the reduced level of O_2_^−^ may lead to increase in NO^·^ bioavailability. This might be explained by the fact that the affinity of NO^·^ issued by eNOS is greater in acting with O_2_^−^ within the endothelial cell compared to its affinity with the spin trap used, namely, DETC. Thus, NO^·^ may be blunted by the O_2_^−^ release to form ONOO^−^. This hypothesis is reinforced by the potent antioxidant properties of LMAE *in vitro*, comparable to those of the reference control, Trolox.

The mechanism by which LMAE decreased oxidative stress was further examined by assessing changes in the expression of endogenous prooxidant enzymes in the arterial wall. Among these enzymes, different reports had shown the importance of COX-2 and NADPH oxidase in the endothelial dysfunction, oxidative stress, and progression of Ang II-induced hypertension [23]. Indeed, COX-2 activation induced the release of COX-derived vasoconstrictor metabolites [32]. Also, in the arterial wall of rats, both membrane-bound NADH/NADPH oxidase activity and the expression of different several NADPH oxidase subunits were increased [31]. Using the experimental model similar to the present study, the concomitant overproduction of reactive oxygen species from NADPH oxidase and/or mitochondria and the activation of COX-2/TP receptor pathway provoked vascular dysfunction including endothelial dysfunction, increased vascular reactivity, and hypertension [23]. In the present study, LMAE treatment reduced the expression of both the inducible isoform of cyclooxygenase, COX-2, and the NADPH oxidase isoform NOX-2 of the mouse aortas. The antioxidant property of LMAE could act in synergy with its ability to regulate the expression of prooxidant enzyme to mitigate the damaging effects of oxidative stress and exert vasculoprotection [33, 34]; such mechanism could be supporting the antihypertensive effect of LMAE.

Supporting this hypothesis, it is known that hypertension is associated with increased vascular reactivity to vasoconstrictor agents. Increased cytosolic calcium, *via* either voltage-dependent calcium channels, receptor-dependent calcium entry, or release of Ca^2+^ from intracellular stores, in addition to calcium sensitization of contractile proteins participates to vascular hyperactivity observed in hypertension. Notably, the contractile response to agonist relative to calcium entry is primarily due to voltage-dependent calcium channels beside the involvement of calcium entry *via* receptor-operated calcium channels [35, 36]. In the present study, LMAE completely prevented CaCl_2_-induced contraction in the KCl-exposed mice aorta *ex vivo*. Thus, LMAE might exert its protective effects *via* inhibition of calcium entry in response to vasoconstrictor agonists at the level of smooth muscle cells in addition to its action on the endothelium.

Finally, although further studies are needed to characterize the compounds that support the antihypertensive properties of LMAE on the vascular wall, the phytochemical analysis of LMAE extracts showed that it contained sterols, triterpenes, coumarins, and anthraquinone. All of these compounds were previously described to have antioxidant and vasodilator properties [37–40].

## 5. Conclusion

This report is the first report demonstrating that LMAE corrects angiotensin II-induced hypertension and endothelial dysfunction in the aorta in an *in vivo* model. Furthermore, we decipher the mechanisms involved in hypertension correction: reduction of COX-2- and NOX-2-induced oxidative stress and inhibition of calcium entry. Therefore, the present study represents a pharmacological basis of the empirical use of *Lannea microcarpa* trunk bark extract against hypertension.

## Figures and Tables

**Table 1:** Effect of LMAE on echocardiography parameters in an Ang II-induced hypertension model.

	NaCl	NaCl+LMAE	Ang II	Ang II+LMAE
Diastolic diameter (mm)	4.4 ± 0.4	4.5 ± 0.2	4.4 ± 0.3	4.4 ± 0.3
Systolic diameter (mm)	3.2 ± 0.3	3.3 ± 0.2	3.0 ± 0.4	3.2 ± 0.4
Stroke volume (mL)	46.2 ± 8.6	51.7 ± 8.0	51.8 ± 5.3	48.7 ± 6.5
Cardiac output (mL/min)	36.0 ± 17.6	22.1 ± 4.2	27.5 ± 16.6	22.6 ± 5.9
Ejection fraction (%)	53.6 ± 3.9	54.4 ± 5.6	59.8 ± 7.3	54.7 ± 6.8
Shortening fraction (%)	27.6 ± 2.4	28.2 ± 3.9	32.2 ± 5.0	28.4 ± 4.2
Diastolic LVAW (mm)	0.9 ± 0.1	1.0 ± 0.2	0.9 ± 0.1	0.9 ± 0.3
Diastolic LVPW (mm)	0.9 ± 0.3	1.0 ± 0.1	1.0 ± 0.1	1.1 ± 0.2
Systolic LVAW (mm)	1.3 ± 0.1	1.5 ± 0.3	1.4 ± 0.2	1.4 ± 0.4
Systolic LVPW (mm)	1.1 ± 0.1	1.2 ± 0.1	1.3 ± 0.1	1.3 ± 0.2

LMAE (50 mg/kg) was administered daily by oral gavage for 3 weeks, and osmotic minipumps delivering NaCl (0.9%) or angiotensin II (0.5 mg/kg/day) were implanted 1 week after the start of the LMAE pretreatment. Cardiac function was evaluated by echocardiography. This table shows the systolic and diastolic diameters, stroke volume, cardiac output, ejection and fractions, and left ventricle posterior and anterior wall (LVPW, LVAW) thickness in the systolic and diastolic phase. NaCl: mice treated with NaCl, control; NaCl+LMAE: mice treated with LMAE and NaCl; Ang II: mice treated with Ang II; Ang II+LMAE: mice treated with LMAE and Ang II. NaCl and Ang II groups were treated with an equal volume of vehicle (2% of Tween 80). The results are expressed as mean ± SEM. *n* = 8/group.

**Table 2:** Qualitative phytochemical analysis of trunk bark extracts from *Lannea microcarpa*.

Plant solvent extraction	Phytochemical groups										
	Flavonoid aglycone	Emodols	Sterols and triterpenes	Alkaloids	Coumarins	Flavonoids	Anthraquinone	Tannins	Saponins	anthocianosides	Reducing compounds
DCM	-	-	+	-	-	*nd*	*nd*	*nd*	*nd*	*nd*	*nd*
AE	*nd*	*nd*	+	*nd*	+	-	+	*nd*	*nd*	*nd*	*nd*
aq	*nd*	*nd*	*nd*	*nd*	*nd*	*nd*	*nd*	+	+	+	+

LMDCM: dichloromethane; AE: ethyl acetate; aq: aqueous decoction. Keys: + = present; - = absent; nd = not determined.

## Data Availability

The data used to support the findings of this study are included within the article.

## Supplementary Materials

Supplementary Materials Supplemental Figure 1: heart and kidney weights and kidney parameters. Mean relative heart (A) and kidney (B) weights after 21 days of treatment with LMAE and Ang II. Plasma levels of sodium (Na^+^) (C), chloride (Cl^−^) (D), urea (E), and creatinine (F). The results are expressed as mean ± SEM. *n* = 7-8/group. Supplementary Figure 2: TLC plate images from ethyl acetate fraction of L. microcarpa trunk barks. Photographs represent anthracenosides, coumarins, saponins, triterpenoids and sterols, and tannins, respectively. Silica gel 60 F254 plates; LMAE: ethyl acetate fraction; LMAE': ethyl acetate fraction hydrolysed by acid chlorhydrique 10% solution.
