# Supplementary material for: Ethyl Acetate Fraction of Lannea microcarpa Engl. and K. Krause (Anacardiaceae) Trunk Barks Corrects Angiotensin II-Induced Hypertension and Endothelial Dysfunction in Mice
Source: Oxid Med Cell Longev. 2019 Apr 28;2019:9464608. doi: 10.1155/2019/9464608 (PMC6512010; doi:10.1155/2019/9464608)
Supplement: Supplementary Materials — Supplemental Figure 1: heart and kidney weights and kidney parameters. Mean relative heart (A) and kidney (B) weights after 21 days of treatment with LMAE and Ang II. Plasma levels of sodium (Na+) (C), chloride (Cl−) (D), urea (E), and creatinine (F). The results are expressed as mean ± SEM. n = 7-8/group. Supplementary Figure 2: TLC plate images from ethyl acetate fraction of L. microcarpa trunk barks. Photographs represent anthracenosides, coumarins, saponins, triterpenoids and sterols, and tannins, respectively. Silica gel 60 F254 plates; LMAE: ethyl acetate fraction; LMAE': ethyl acetate fraction hydrolysed by acid chlorhydrique 10% solution. [file 9464608.f1.pdf]

Surgical procedure for osmotic minipums implantation

Osmotic minipumps (Model 1002, Alzet Osmotic Pumps, Cupertino, CA) containing Ang II or NaCl were subcutaneously implanted into the back of mice. The animals were weighted and then, anesthesia was induced in the induction boxes, with 5% isoflurane at air flow rate of 0.2 L/min. Once the animal felt asleep, it was placed on a heating pad to keep the temperature at 37°C. Throughout the surgical procedure the anesthesia with isoflurane was maintained (2% isoflurane under air flow). A dose of buprenorphine (0.1 mg / kg) was subcutaneously injected. Minipumps was implanted subcutaneously on the back of mice, slightly posterior to the scapulae. The animals were shaved, and then the povidone iodine was applied on the treated zone. An incision 1 cm was then made. The subcutaneous tissue was discarded to create a pocket to insert the minipump. Once the minipumps was inserted, the wound was closed by wound clips. Buprenorphine (0.1 mg / kg) was subcutaneously injected for pain relieve and anesthesia was stopped. Mice were covered with heated blankets to maintain body temperature till animals completely recovered from anesthesia. Surgical site was monitored daily to ensure the presence of intact wound clips and for any abnormal wound discharge or infection. Body weight, animal activity and any abnormal changes in activity/behavior were noted.

Supplemental figure 1

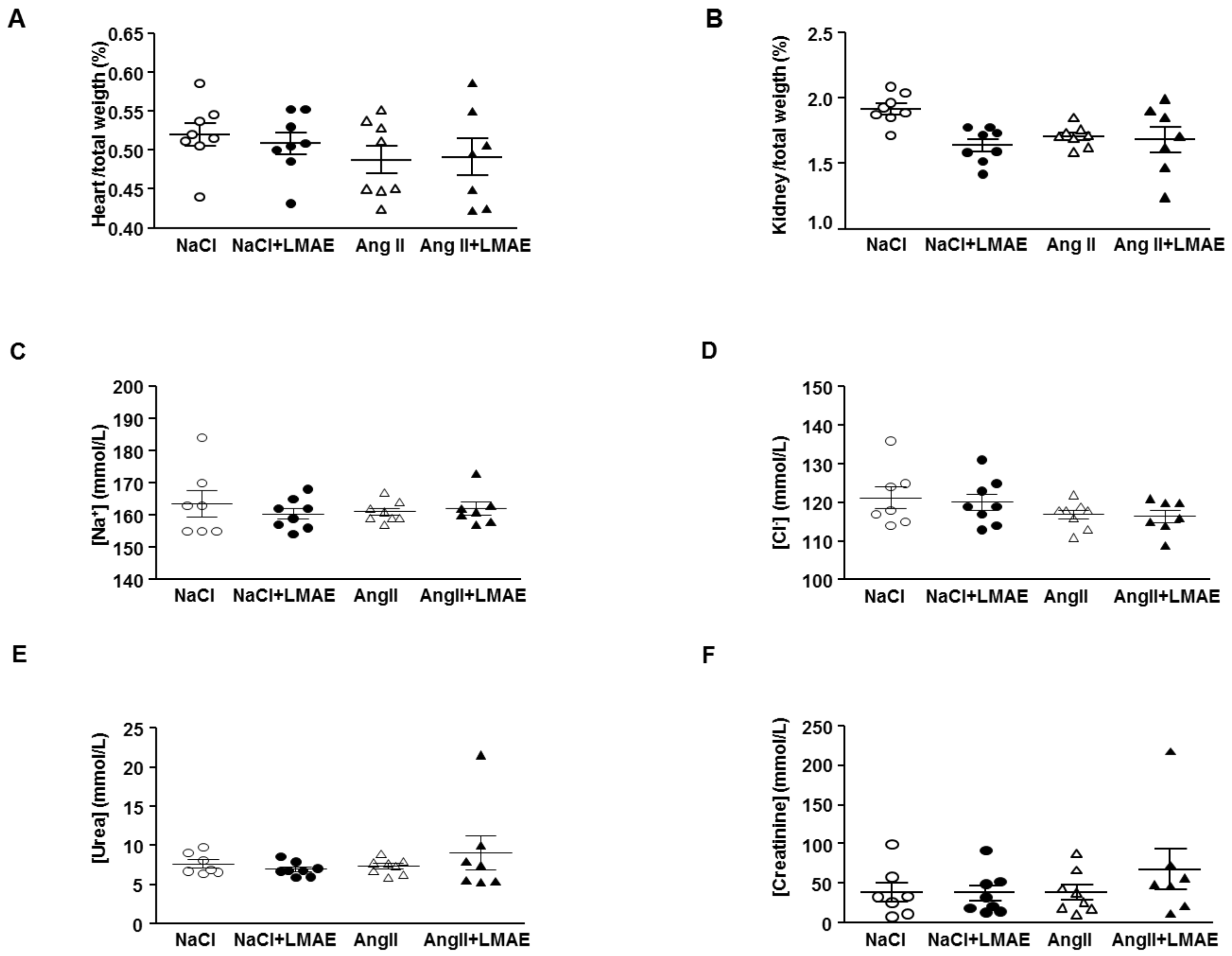

Supplemental figure 1. Heart and kidney wights and kidney parameters. Mean relative heart (A) and kidneys (B) weights of after 21 days treatment with LMAE and Ang II. Plasma levels of sodium (Na+) (C), chlore (Cl^-) (D), urea (E) and creatinine (F). The results are expressed as mean ± SEM. n=7-8/group.

Supplemental figure 2

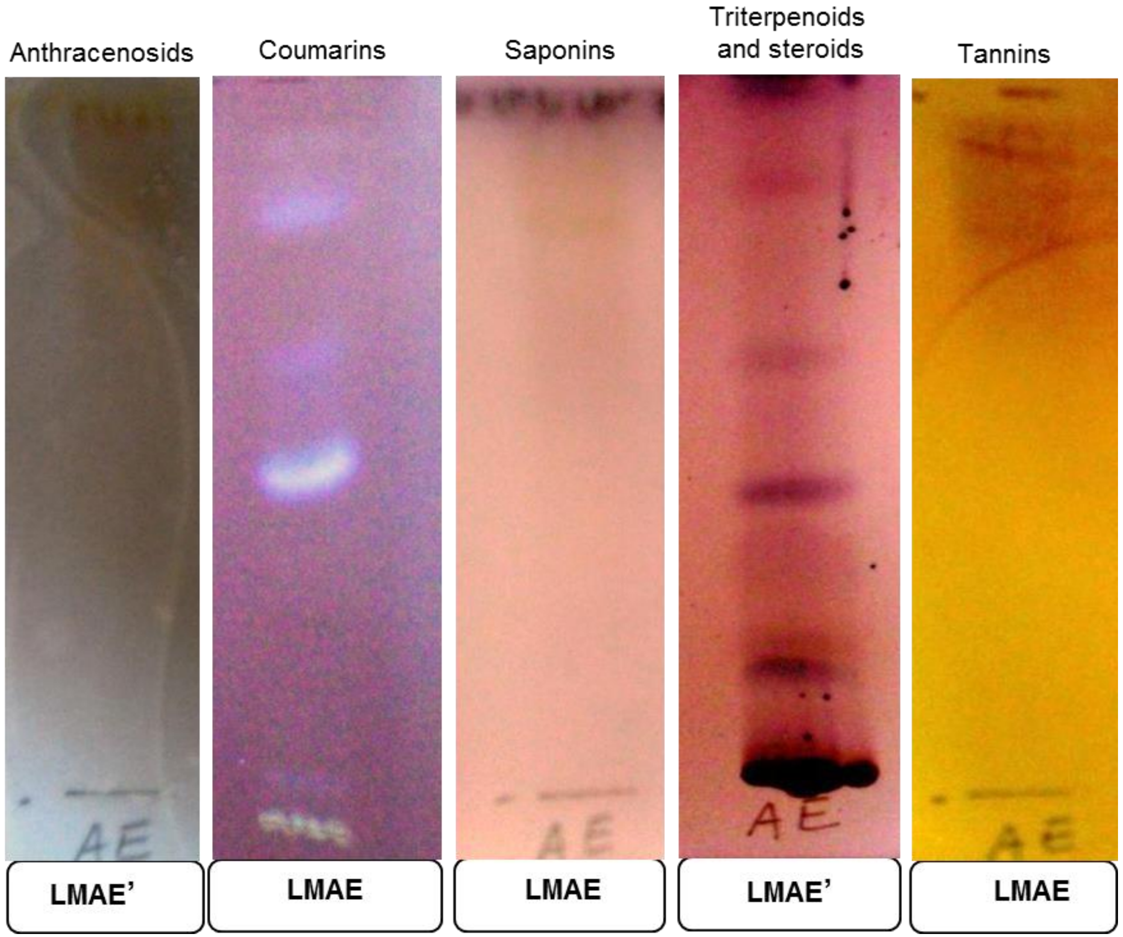

Supplementary Figure 2. TLC plates images from ethyl acetate fraction of *L. microcarpa* trunk barks. Photographs represent respectively anthracenosides, coumarins, saponins, triterpenoids and sterols and tannins.bSilica gel 60 F_{254} plates; LMAE: ethyl acetate fraction; LMAE': ethyl acetate fraction hydrolsed by acid chlorhydrique 10% solution.
